# Trichophyton rubrum Skin Folliculitis in Behçet's Disease

**DOI:** 10.7759/cureus.20349

**Published:** 2021-12-11

**Authors:** Yassine Merad, Malika Belkacemi, Hichem Derrar, Nafissa Belkessem, Samira Djaroud

**Affiliations:** 1 Central Laboratory, Parasitology-Mycology, Hassani Abdelkader University Hospital, Sidi Bel Abbès, DZA; 2 Hemobiology and Blood Transfusion, Hassani Abdelkader University Hospital, Sidi Bel Abbès, DZA; 3 Pulmonary and Lung Diseases, Hassani Abdelkader University Hospital, Sidi Bel Abbès, DZA; 4 Botanic Department, Department of Epidemiology and Preventive Medicine, Sidi Bel Abbès University Hospital, Sidi Bel Abbès, DZA; 5 Chemistry Department, Djillali Liabes University (UDL), Sidi Bel Abbès, DZA

**Keywords:** skin disease, trichophyton rubrum, dermatophytosis, folliculitis, behçet’s disease

## Abstract

A 29-year-old patient with Behçet’s disease based on three major criteria (i.e., oral ulceration, genital ulceration, and eye lesion) presented with intractable pruritus associated with pinpoint red nodules involving the hair follicles of the back along with steroid-refractory local treatment. Simple light microscopic examination of a skin scraping revealed fungal contamination, and culture on Sabouraud’s medium confirmed *Trichophyton rubrum* as the agent of folliculitis. Behçet’s disease is characterized by recurrent attacks of acute inflammation. Although the diagnosis of sterile folliculitis-like disorder is currently retained among patients with Behçet’s disease, especially in the lower part of the body, it resembles dermatophytic folliculitis, which can be related to immunosuppressive therapy. Hence, patients with recalcitrant folliculitis predominating on the back who are receiving immunosuppressive treatment should be evaluated for fungal infection, as recognition of this disease may enable earlier diagnosis and treatment.

## Introduction

Behçet’s disease (BD) is a multisystemic inflammatory vasculitic disorder with a predilection for the skin of the mouth, eye, and genitals. Because of the lack of specific laboratory tests and/or pathognomonic histopathological findings, this condition is diagnosed via clinical criteria [1].

Patients with BD have pustular skin lesions that resemble acne but can occur nearly anywhere on the body and particularly on the lower extremities. This rash is sometimes called “folliculitis,” and the lesions may be clinically and histopathologically indistinguishable from acne vulgaris [1,2].

Making a chloral-lactophenol preparation from the active or leading-edge scraping optimizes the diagnosis of dermatophytosis, and culture or biopsy with periodic acid−Schiff staining is useful in equivocal cases. Nevertheless, dermatophytosis is difficult to diagnose in immunosuppressed patients, because the clinical presentation is often atypical [3].

*Trichophyton rubrum* Majocchi’s granuloma is an uncommon deep fungal infection that may develop in any hair-bearing skin but is mostly observed in the lower extremities. The second form of this disease is granulomatous, related to immunosuppression, seen in a nodular form, and usually appears on the upper extremities [4]. The presence of invasive *Epidermophyton floccosum *has been reported in an immunocompromised patient with BD and proven by biopsy in an immunocompromised patient [5,6].

## Case presentation

We report the case of a 29-year-old male patient who had a two-year history of BD based on an initial manifestation with oral ulcers with episodes of recurrent, bilateral, nongranulomatous chronic uveitis. The patient was taking systemic corticosteroids, topical steroids, and nonimmunosuppressive medication, colchicine (one tablet daily), and azathioprine (2 mg/kg daily).

**Figure 1:**
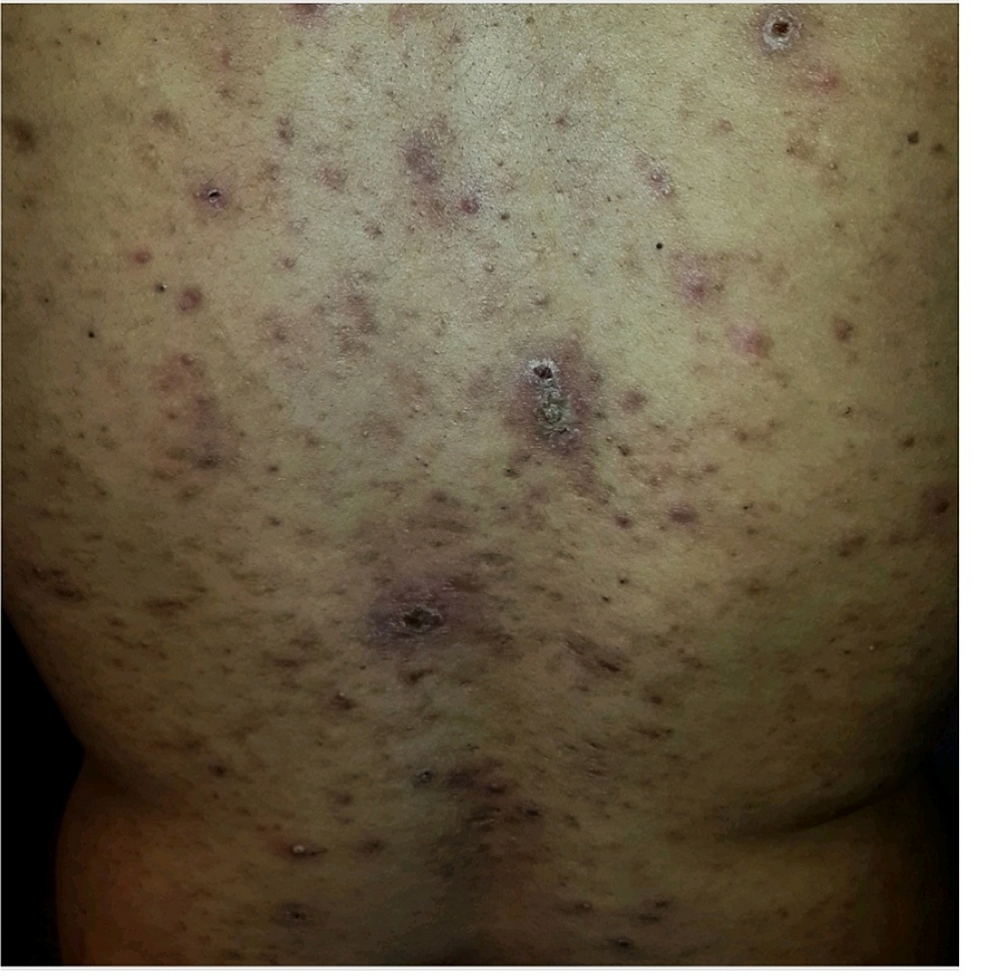
folliculitis of the back

On physical examination, the patient had the accepted criteria of BD, painful sores in the mouth referred to as “aphthous ulcers,” genital ulcers, and folliculitis on the back (Figure 1).

The patient was initially managed with several courses of topical steroids to reduce the inflammation of the back. However, because of the lack of significant improvement, he was referred to our hospital for further investigation. Back lesions were noted to be sterile, resembled folliculitis, and had an acne-like appearance (Figure 1).

BD is characterized by recurrent oral aphthous ulcerations, genital ulcerations, mucocutaneous manifestations, and uveitis. Oral ulcers are classified by size and are categorized as minor (<1 cm), major, and herpetiform ulcers. Our patient had painful minor ulcers with well-defined borders with erythematous halo. Mucocutaneous lesions appeared at the onset of and during the course of the disease.

The criteria for dermatologic findings in BD include pseudo-folliculitis, acneiform nodules, and papulopustular lesions. In our patient, the second most affected site was the scrotum and the foreskin. Lesions on the back had an acne-like appearance.

**Figure 2:**
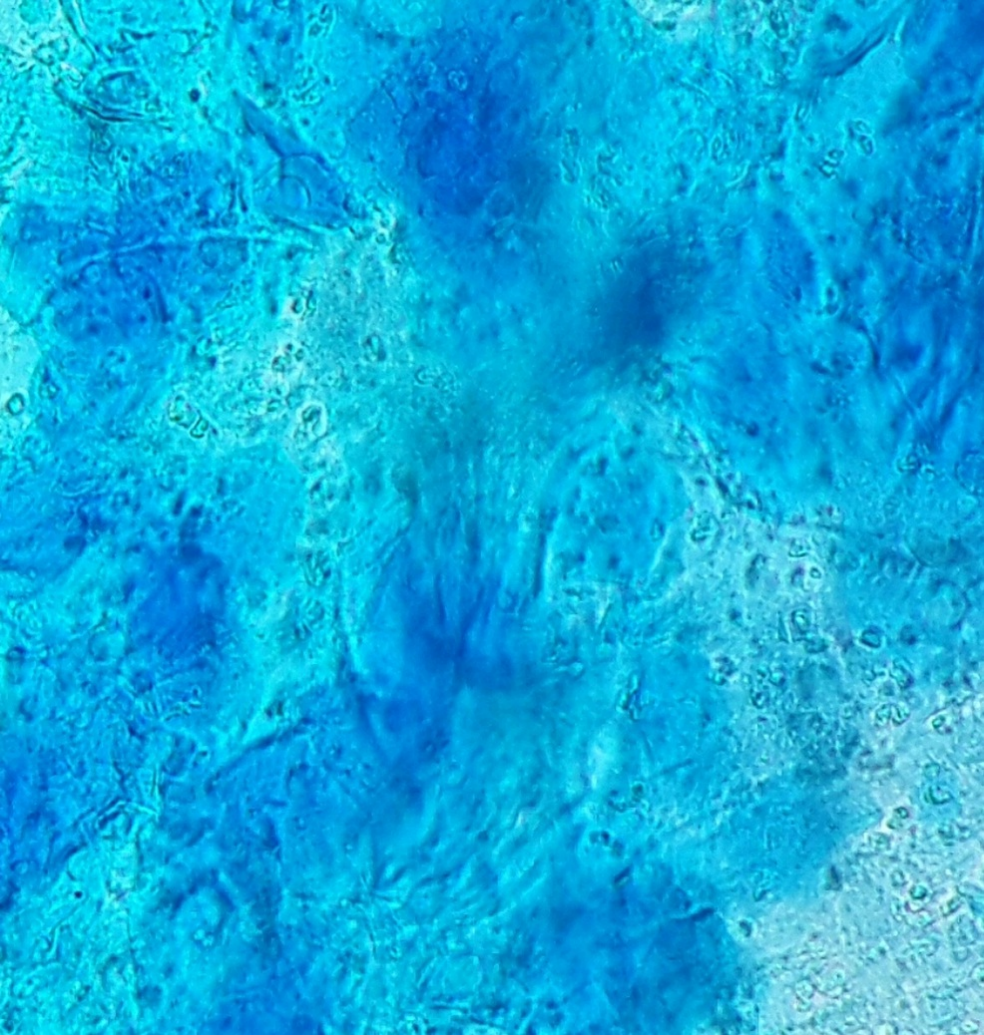
Microscopic examination of skin scraping, revealing fungal elements

The results of routine blood tests were within the normal ranges, and C-reactive protein was not elevated. Anti-HIV and total hepatitis B core antibody (anti-HBc) tests were negative. Abundant fungal elements were seen in chloral-lactophenol testing under direct microscopic examination of the back lesion. Moreover, there were no clinical signs of fungal infection on hands and nails (Figure 2).

**Figure 3:**
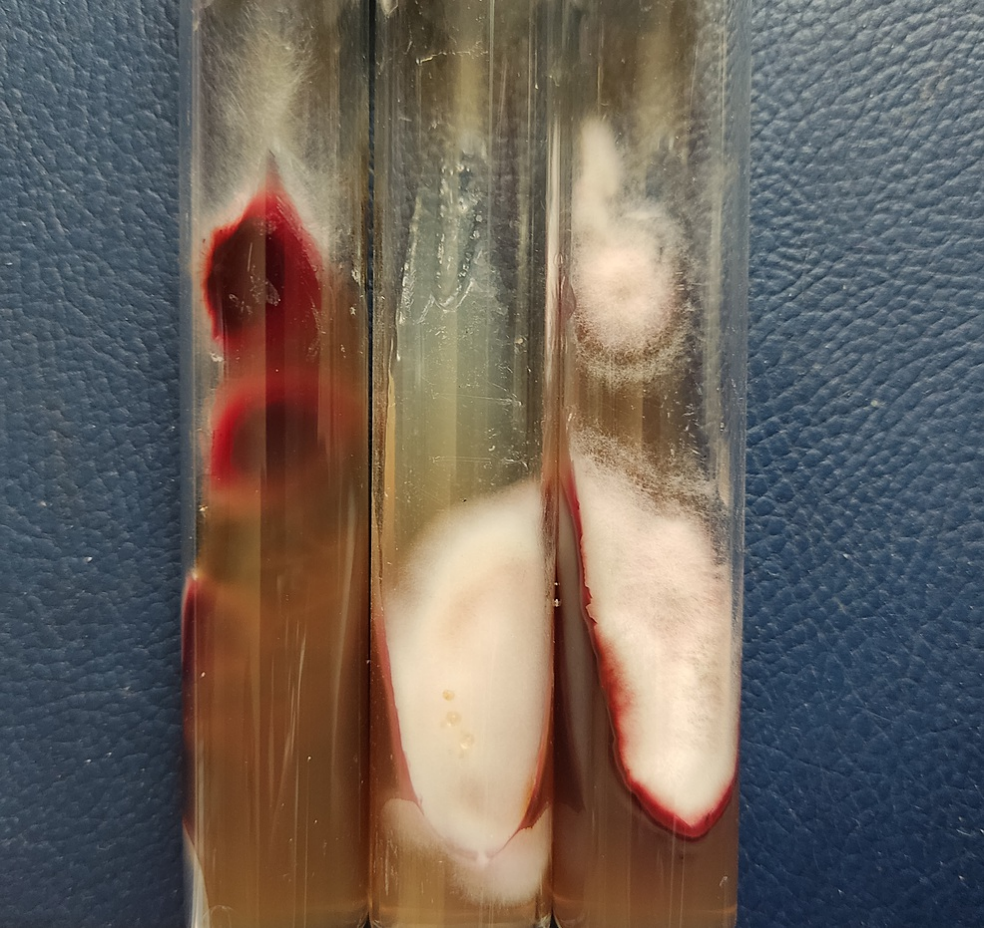
Trichophyton rubrum culture on Sabouraud dextrose agar (SDA) medium

Results of Sabouraud’s medium culture were compatible with *Trichophyton* *rubrum *(Figure 3).

The management of BD is long and often difficult. Baseline treatment for our patient consisted of a combination of colchicine, corticosteroids, and azathioprine.

Our patient was started on itraconazole therapy 200 mg/day for one week each month, repeating the monthly cycle three times, in addition to ciclopirox olamine cream applied twice daily for four weeks. Additional measures included good hygiene and handwashing, keeping the skin clean and dry, showering after engaging in contact sports, and not sharing personal care items. The patient was instructed to avoid using sponges for the bath or shower, sharing razors, towels, and washcloths, and attending public swimming facilities, hot tubs, spas, and similar facilities during treatment.

The patient experienced improvement during the first month and reported no itching. Only a few lesions remained at the last follow-up, during the second month.

## Discussion

Folliculitis is a common skin condition that occurs when a hair follicle becomes infected or inflamed by parasites, viruses, bacteria, or chronic disease. Folliculitis eruptions have a broad clinical spectrum and differ by location, morphology, and etiological agent. Table 1 depicts the causes of folliculitis causes, and Table 2 summarizes the different locations and etiological infectious agents of folliculitis.

**Table 1:** Folliculitis and folliculitis-like etiologies

Folliculitis/folliculitis-like etiologies	Specific characteristics
►Parasitic etiologies: scabiose	Contagious (family cases), itching mainly at night, rash causes little bumps that often form a line, involving many parts of the skin, around the nipples, buttocks, between the fingers, elbows, wrists, and penis
►Hormonal/genetic/autoimmune etiologies: eosinophilic folliculitis, acne vulgaris, steroid acne	Common among teenagers, involving the face and neck, crusting of skin bumps, papules, pustules, painful cystic lesions, redness around the skin eruption, comedones
►Bacterial etiologies	Lack of hygiene, papules, and pustules occur in crops; they are painless but may be pruritic. Favored sites include the scalp, beard areas, axilla, buttocks, and extremities
►Fungal etiologies : dermatophytosis, malassezia folliculitis	Majocchi’s granuloma (dermatophytic folliculitis) of the legs, arms, and ankles Patients with malassezia folliculitis are usually adolescent and young adult males. Itchy papules and pustules occur on the upper back and chest. Other involved sites can include the forehead/hair line, chin, and neck.
►Viral etiologies: varicella–zoster, Molluscum contagiosum	Furunculosis consists of painful, burning, red grouped or scattered fluid-filled blisters; they are refractory to anti-infective and anti-inflammatory treatment. Patients with molluscum folliculitis present with multiple discrete whitish papules.

**Table 2:** Folliculitis by location and microorganisms

Folliculitis by location	Microorganisms reported
►Face	Simplex virus, Molluscum contagiosum, Trichophyton rubrum, Trichophyton mentagrophyte, Demodex sp., Propionibacterium granulosum coagulase negative staphylococci, a-hemolytic streptococci, Staphylococcus aureus, Escherichia coli, Provotella sp.
►Back, chest	Simplex virus, Malassezia sp., T. rubrum, Epidermophyton floccosum, Propionibacterium granulosum, coagulase negative staphylococci, a-hemolytic streptococci, S. aureus, Provotella sp.
►Legs	T. rubrum, S. aureus, Provotella sp.
►Arms	T. rubrum, S. aureus, Provotella sp.

Sterile folliculitis is the current retained diagnosis in BD patients. The distribution of pustules from patients with BD on the arms, legs, back, and face is important [1,7]. These lesions are sterile and may resemble folliculitis or have an acne-like appearance. One clue to differentiation between BD folliculitis-like lesions and acne lesions is the absence of comedones and marked involvement of the extremities [2].

However, features that favor the diagnosis of BD include the number of ulcers (>six), synchronous occurrence of more than one clinical variant, diffuse exanthem, and soft palate, and oropharyngeal involvement. Oral ulcers in patients with BD can leave scars and sequelae, such as dysphagia, odynophagia, and dyspnea. Clinically, these sequelae are practically indistinguishable from recurrent oral aphthosis.

*T. rubrum* is an anthropophilic fungi. Autoinoculation via back scratching from infected nails is not a rare phenomenon [8,9], but there was no evidence of clinical onyxis in our patient. However, the condition can also be associated with fomites [10,11]. Occasionally, tinea corporis infections are zoonotic, and may be related to farm work with stock animals. Our patient lived in an urban area and reported no contact with animals.

Majocchi’s granuloma presents with small skin lesions and inflamed hair follicles in areas that are prone to trauma, such as the legs, arms, and ankles. Meanwhile, individuals with a decreased immune response may present with more severe manifestations, such as deep subcutaneous plaques and nodules [5,9,10].

The combination of our findings was highly suspicious for dermatophytic folliculitis, especially because the skin mycological tests were compatible with *T. rubrum* and the light microscopy examination of the skin scraping revealed a fungal presence. Otherwise, histologic evaluation can also aid in the detection of other forms of folliculitis.

BD lesions are sterile and may resemble folliculitis or have an acne-like appearance. However, follicles in BD can be infected, and it remains to be determined whether these pustules are infected secondarily or whether the infections play a pathogenic role in the development of pustular lesions, and possibly of arthritis [7].

Dermatophyte folliculitis appears as pinpoint red nodules, each of which involves a hair follicle. *T. rubrum* folliculitis can appear on a herpes zoster scar [12], and it is possible that *T. rubrum *can develop folliculitis-like BD, sterile folliculitis-like lesion containing bacteria was already reported [7].

Immunosuppressive agents used in the treatment of BD include azathioprine, tacrolimus, cyclosporine, and a combination of prednisone and cyclophosphamide. These drugs may promote fungal growth [3].

Dermatophytosis is usually observed after autoinoculation from local trauma caused by a scratch or, more commonly, by shaving razors. Fomites such as towels, brushes, hats, and upholstery can be important in transmission. Once the infectious elements of the fungus (arthroconidia) enter the skin, they germinate and invade the superficial skin layers. The infectivity and inflammatory potentials vary by species and can be facilitated by obesity, perspiration, humidity, and heat [13,14].

*T. rubrum* is the species most involved in severe forms of folliculitis; disseminated dermatophytosis was described in a patient with advanced cirrhosis [13].

In immunocompromised patients, severe forms of BD include extensive and/or invasive dermatophytosis (i.e., deep dermatophytosis and Majocchi’s granuloma) [4]. Moreover, alterations in the normal skin microflora from previous or concurrent therapy play a role as predisposing factors, in that they allow the overgrowth of fungal organisms, especially dermatophytes [3,9,10,11].

## Conclusions

Corticosteroids and immunosuppressive treatments used for BD are more likely to induce dermatophytosis.* T. rubrum *folliculitis of the back should be differentiated from the current sterile folliculitis-like condition in BD using mycological investigation, especially in the presence of intractable itching alongside poor adherence to local anti-inflammatory therapy.
